# Supplementary material for: Concomitant Infection of Helicobacter pylori and Intestinal Parasites in Adults Attending a Referral Centre for Parasitic Infections in North Eastern Italy
Source: J Clin Med. 2020 Jul 24;9(8):2366. doi: 10.3390/jcm9082366 (PMC7465117; doi:10.3390/jcm9082366)
Supplement: Supplementary file 1 [file jcm-09-02366-s001.zip › suppl files/suppl files/Table S1.docx]

**Table S1.** Primer/probe sets of four multiplex real-time PCR for intestinal parasites

| **Multiplex** | **Parasite** | **Primers/probe** | **Ref** |
| --- | --- | --- | --- |
| 1 | *Entamoeba histolytica* | For 5’-ATTGTCGTGGCATCCTAACTCA-3’  Rev 5’-GCGGACGGCTCATTATAACA-3’  Probe 5’-FAM-TCATTGAATGAATTGGCCATTT-3’-MGB | 33 |
|  | *Entamoeba dispar* | For 5’-ATTGTCGTGGCATCCTAACTCA-3’  Rev 5’-GCGGACGGCTCATTATAACA-3’  Probe 5’-HEX-TTACTTACATAAATTGGCCACTTTG-3’-MGB | 33 |
|  | *Cryptosporidium spp.* | For 5’-ATGAGCGGGTAACGGGGAAT-3’  Rev 5’-CCAATTACAAAACCAAAAAGTCC-3’  Probe 5’-CY55-CGCGCCTGCTGCCTTCTTTAGATG-3’-BBQ | 34 |
| 2 | *Giardia intestinalis* | For 5’-GACGGCTCAGGACAACGGTT-3’  Rev 5’-TTGCCAGCGGTGTCCG-3’  Probe 5’-CY55-CCCGCGGCGGTCCCTGCTAG-3’-BHQ | 35 |
|  | *Dientamoeba fragilis* | For 5’-CAACGGATGTCTTGGCTCTTTA-3’  Rev 5’-TTGCCAGCGGTGTCCG-3’  Probe 5’-HEX-CAATTCTAGCCGCTTAT-3’-MGB | 36 |
|  | *Blastocystis spp.* | For 5’-GGTCCGGTGAACACTTTGGATTT-3’  Rev 5’-CCTACGGAAACCTTGTTACGACTTCA-3’  Probe 5’-FAM-TCGTGTAAATCTTACCATTTAGAGGA-3’-MGB | 37 |
| 3 | *Strongyloides stercoralis* | For 5’-GAATTCCAAGTAAACGTAAGTCATTAGC-3’  Rev 5’-TGCCTCTGGATATTGCTCAGTTC-3’  Probe 5’-FAM-ACACACCGGCCGTCGCTGC-3’-BHQ | 38 |
|  | *Schistosoma spp* | For 5’-GGTCTAGATGACTTGATYGAGATGCT-3’  Rev 5’-TCCCGAGCGYGTATAATGTCATTA-3’  Probe 5’-FAM-TGGGTTGTGCTCGAGTCGTGGC-3’-BHQ | 39 |
|  | *Hymenolepis nana* | For (Hna-F1) 5’-CATTGTGTACCAAATTGATGATGAGTA-3’  Rev (Hna-R1) 5’-CAACTGACAGCATGTTTCGATATG-3’  Probe (Hna-1-probe)  5’-YAKYE-CGTGTGCGCCTCTGGCTTACCG-3’-BHQ | Developed by Dr Verweij JJ |
| 4 | *Necator americanus* | For 5’-CTGTTTGTCGAACGGTACTTGC-3’  Rev 5’-ATAACAGCGTGCACATGTTGC-3’  Probe 5’-FAM-CTGTACTACGCATTGTATAC-3’-MGB | 40 |
|  | *Ascaris lumbricoides* | For 5’-GTAATAGCAGTCGGCCGGTTTCTT-3’  Rev 5’-GCCCAACATGCCACCTATTC-3’  Probe 5’-ROX-TTGGCGGACAATTGCATGCGAT-3’-BHQ | 40 |
|  | *Ancylostoma duodenale* | For 5’-GAATGACAGCAAACTCGTTGTTG-3’  Rev 5’-ATACTAGCCACTGCCGAAACGT-3’  Probe 5’-HEX-ATCGTTTACCGACTTTAG-3’-MGB | 40 |
|  | *Trichuris trichiura* | For 5’-TCCGAACGGCGGATCA-3’  Rev 5’-CTCGAGTGTCACGTCGTCCTT-3’  Probe 5’-CY55-CGATGGTACGCTACGTGCTTACCATGG-3’-BHQ | 41 |
