# Supplementary material for: Concomitant Infection of Helicobacter pylori and Intestinal Parasites in Adults Attending a Referral Centre for Parasitic Infections in North Eastern Italy
Source: J Clin Med. 2020 Jul 24;9(8):2366. doi: 10.3390/jcm9082366 (PMC7465117; doi:10.3390/jcm9082366)
Supplement: Supplementary file 1 [file jcm-09-02366-s001.zip › suppl files/suppl files/Table S2.docx]

**Table S2.** Baseline characteristics of the subjects positive to *H. pylori* (detected by SAT) and screened for *cagA* (detected by rt-PCR). Categorical and continuous variables are presented as numbers (%) and medians (interquartile range), respectively.

| **Variable** | | ***cagA+***  **(N=31)** | ***cagA-***  **(N=30)** | ***p* value** |
| --- | --- | --- | --- | --- |
| Age (years) |  | 24 (20-35) | 31 (23-40) | 0.0922 |
| Sex | Female  Male | 1 (11.11)  30 (57.69) | 8 (88.89)  22 (42.31) | 0.0105  -  - |
| Geo Origin | Africa | 29 (54.72) | 24 (45.28) | - |
|  | Italy | - | 2 (100) | - |
|  | Asia | - | 2 (100) | - |
|  | South-America | 1 (50) | 1 (50) | - |
|  | East-Europe | 1 (50) | 1 (50) | - |
| Clinical features | Abdominal pain | 3 (51.85) | 4 (57.14) | 0.2824 |
|  | Epigastric pain | 2 (25) | 6 (75) | 0.0938 |
|  | Diarrhea | 2 (66.67) | 1 (33.33) | 0.3876 |
| Endoscopy findings |  |  |  | 0.1091 |
|  | Chronic gastritis | 1 (100) | - |  |
|  | Chronic gastritis and erosive duodenitis | - | 1 (100) |  |
|  | Antral gastritis and bulbar duodenitis | - | 2 (100) |  |
