# Supplementary material for: Concomitant Infection of Helicobacter pylori and Intestinal Parasites in Adults Attending a Referral Centre for Parasitic Infections in North Eastern Italy
Source: J Clin Med. 2020 Jul 24;9(8):2366. doi: 10.3390/jcm9082366 (PMC7465117; doi:10.3390/jcm9082366)
Supplement: Supplementary file 1 [file jcm-09-02366-s001.zip › suppl files/suppl files/Table S3.docx]

**Table S3.** Baseline characteristics of the subjects positive to *H. pylori* (Hp) and screened for intestinal parasites (IP). Categorical and continuous variables are presented as numbers (%) and medians (interquartile range), respectively.

| **Variable** | | **Hp+/IP+**  **(N=45)** | **Hp+/IP-**  **(N=16)** | ***p* value** |
| --- | --- | --- | --- | --- |
| Age (years) |  | 25 (21-35) | 33 (24.50-41) | 0.1438 |
| Sex | Female  Male | 7 (77.78)  38 (73.08) | 2 (22.22)  14 (26.92) | 0.3140  -  - |
| Geo Origin | Africa | 40 (75.47) | 13 (24.53) | - |
|  | Italy | 2 (100) | - | - |
|  | Asia | 2 (100) | - | - |
|  | South-America | - | 2 (100) | - |
|  | East-Europe | 1 (50) | 1 (50) | - |
| Clinical features | Abdominal pain | 6 (72.22) | 1 (14.29) | 0.2186 |
|  | Epigastric pain | 5 (62.50) | 3 (37.50) | 0.2987 |
|  | Diarrhea | - | 3 (100) | 0.2323 |
| Endoscopy findings |  |  |  | 0.0156 |
|  | Chronic gastritis | 1 (100) | - |  |
|  | Chronic gastritis and erosive duodenitis | - | 1 (100) |  |
|  | Antral gastritis and bulbar duodenitis | - | 2 (100) |  |
