# Supplementary material for: Concomitant Infection of Helicobacter pylori and Intestinal Parasites in Adults Attending a Referral Centre for Parasitic Infections in North Eastern Italy
Source: J Clin Med. 2020 Jul 24;9(8):2366. doi: 10.3390/jcm9082366 (PMC7465117; doi:10.3390/jcm9082366)
Supplement: Supplementary file 1 [file jcm-09-02366-s001.zip › suppl files/suppl files/Table S4.docx]

**Table S4.** Baseline characteristics of the subjects negative to *H. pylori* (Hp) and screened for intestinal parasites (IP). Categorical and continuous variables are presented as numbers (%) and medians (interquartile range), respectively.

| **Variable** | | **Hp-/IP+**  **(N=15)** | **Hp-/IP-**  **(N=17)** | ***p* value** |
| --- | --- | --- | --- | --- |
| Age (years) |  | 43 (23-56) | 38 (28-41) | 0.7196 |
| Sex | Female  Male | 7 (53.85)  8 (42.11) | 6 (46.15)  11 (57.89) | 0.2293 |
| Geo Origin | Africa | 13 (52) | 12 (48) | - |
|  | Italy | 1 (25) | 3 (75) | - |
|  | Asia | 1 (50) | 1 (50) | - |
|  | South-America | - | 1 (100) | - |
|  | East-Europe | - | - | - |
| Clinical features | Abdominal pain | 6 (50) | 6 (50) | 0.2603 |
|  | Epigastric pain | 1 (50) | 1 (50) | 0.2743 |
|  | Diarrhea | 2 (50) | 2 (50) | 0.5141 |
| Endoscopy findings |  |  |  | 0.3971 |
|  | Chronic gastritis | - | - |  |
|  | Chronic gastritis and erosive duodenitis | - | 1 (100) |  |
|  | Antral gastritis and bulbar duodenitis | 1 (50) | 1 (50) |  |
