# Supplementary figures and images for: Concomitant Infection of Helicobacter pylori and Intestinal Parasites in Adults Attending a Referral Centre for Parasitic Infections in North Eastern Italy
Source: J Clin Med. 2020 Jul 24;9(8):2366. doi: 10.3390/jcm9082366 (PMC7465117; doi:10.3390/jcm9082366)

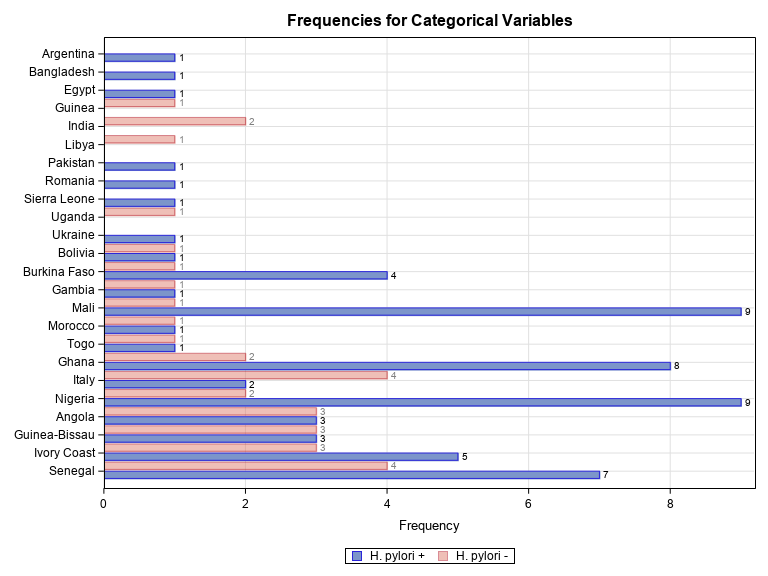

Supplement: Supplementary file 1 [file jcm-09-02366-s001.zip › suppl files/suppl files/Figure S1.tiff]
